# 

**Published:** 1912-03

**Authors:** 


### CONTENTS.

ORIGINAL COMMUNICATIONS—

Etiology, Pathology and Treatment of Pellagra. By Geo. C. Mizell, M. D. ...... 625

Observations and Personal Experience on the Improved Technique of Nitrous-Oxide-Oxygen and the Ether Vapor Anaesthesia. By Thomas J. Collier, M. D., Atlanta, Ga. ...... 643

The Gradual Reduction vs. the Hyoscine or Quick Withdrawal Method in the Treatment of the Morphine Habit. By W. C. Ashworth, M. D., Greensboro, N. C. ...... 648

Pediatrics a Specialty. By Clarence A. Rhodes, A. B., M. D., Atlanta, Ga. .... 656

Mastoiditis: Indications for Operation and Report of Some Interesting Cases. By C. W. Canan, B. S., M. D. Orkney Springs, Va. ...... 660

EDITORIALS ...... 665

To a Tapeworm. By Bernard Wolff, M. D., Atlanta, Ga. ...... 670

SELECTIONS AND ABSTRACTS ...... 672

BOOK REVIEWS ...... 674

